# Comparison of SENSIMED Triggerfish^®^ (TF) 24-Hour Monitoring in Open-Angle Glaucoma Patients Before and After Trabeculectomy

**DOI:** 10.3390/jcm14062112

**Published:** 2025-03-19

**Authors:** Anna Beck, Michael Uhrig, Alexander Schuster, Christina Korb, Norbert Pfeiffer, Katrin Lorenz

**Affiliations:** Department of Ophthalmology, University Medical Center, Johannes Gutenberg-University, 55131 Mainz, Germanyalexander.schuster@unimedizin-mainz.de (A.S.); christina.korb@unimedizin-mainz.de (C.K.); norbert.pfeiffer@unimedizin-mainz.de (N.P.); katrin.lorenz@unimedizin-mainz.de (K.L.)

**Keywords:** glaucoma, trabeculectomy, contact lens sensor, Triggerfish^®^

## Abstract

**Background/Objectives**: In glaucoma patients, the fluctuation in intraocular pressure during the day seems to have an influence on the progression of the disease. The contact lens sensor (CLS) Triggerfish^®^ from the company SENSIMED records ocular dimensional changes for 24 h. The aim was to determine the amplitude of the measurements with a contact lens sensor before and after trabeculectomy (TE) in glaucoma patients. **Methods**: Twelve patients with glaucoma were included in this prospective, single-arm, single-center, interventional study. All patients received a CLS measurement for 24 h 8 ± 2 days before and 12 weeks (±1 week) after surgery. The raw data of the measurements were modeled using a double-harmonic cosine function. Fluctuation in the raw CLS data, amplitude, and the MESOR (midline estimating statistic of rhythm, rhythm-adjusted mean) of the modeled data were investigated. The safety and feasibility of the CLS measurements were evaluated. **Results**: Nine patients underwent the complete 24-h wearing period before and after surgery. Whereas the MESOR changed significantly before and after surgery (*p* = 0.04), the amplitude of the modeled data did not change significantly and the daytime fluctuation and circadian rhythm also did not vary significantly. The CLS could be safely removed from all study eyes after surgery. **Conclusions**: The non-significant change in amplitude suggests that diurnal fluctuations persist after TE. This contrasts with reports in the literature that show that TE reduces diurnal fluctuations. It can be assumed that the significant reduction in the MESOR represents the reduction in the average intraocular pressure over 24 h. The CLS can be used safely three months after TE.

## 1. Introduction

Glaucoma is one of the three most common ophthalmologic diseases besides age-related macular degeneration and diabetic retinopathy.

Elevated intraocular pressure (IOP) represents the main treatable risk factor for the progression of glaucoma disease, for both open-angle glaucoma with high IOP and normal-tension glaucoma [1]. However, not only the level of IOP but also its fluctuation during the day seem to have an influence on the progression of the disease. Detecting this fluctuation using non-continuous tonometry techniques risks missing short-term fluctuation. In contrast, continuous tonometry measures at shorter time intervals and thus provides more information over 24 h.

One method that closely approaches continuous tonometry by recording ocular dimensional changes for 24 h is the contact lens sensor (CLS) Triggerfish^®^ from the company SENSIMED. With the data determined, an analysis of fluctuation, amplitude, and the MESOR can be carried out. Using this sensor, it has already been shown that diurnal fluctuations in CLS measurements can be influenced by daily activities such as walking or cycling and by emotional stress [2]. In addition, this method has been shown to increase the fluctuation in CLS measurements in patients with uveitic glaucoma compared with primary open-angle glaucoma (POAG) [3]. Another study investigated the changes in CLS parameters after trabectome surgery in PEX glaucoma patients. The success of the operation was defined as a reduction in the postoperative IOP value ≤ 21 mmHg and a reduction of ≥20% depending on the preoperative IOP value. A comparison of the success and failure group showed that there was no increase in the nocturnal fluctuation in CLS values in the success group [4]. Another study showed that neither the circadian rhythm nor the amplitude of CLS measurements changed after high-frequency deep sclerotomy (HFDS) in POAG patients [5].

In advanced glaucoma, or if medication is not sufficient or not tolerated, surgical therapy is an option. German ophthalmologists consider trabeculectomy one of the most effective surgical interventions to achieve the target pressure.

The primary endpoint of this study was the comparison of the amplitude of the modeled CLS data before and after TE. The secondary endpoints of this study were the change in the MESOR, the reduction in IOP, and fluctuation in the raw CLS data. Furthermore, we evaluated the feasibility and safety of continuous measurement using the CLS after trabeculectomy.

## 2. Materials and Methods

This study was registered at ClinicalTrials.gov (http://clinicaltrials.gov/ct2/show/NCT04000828, first submitted on 27 May 2019, accessed on 18 March 2025) before the enrollment of the first patient. Recruitment began on 22 July 2019 and ended on 5 January 2021. In this prospective and monocentric trial, patients with primary open-angle glaucoma (POAG), pseudoexfoliation glaucoma (PEXG), pigment-dispersion glaucoma, and normal-tension glaucoma (NTG) were examined with a contact lens sensor before and after trabeculectomy. A 24-h measurement of the study eye using the CLS was performed 8 ± 2 days before and 12 weeks ± 1 week after surgery. All study participants had to be at least 18 years old and able to participate in the study. Patients diagnosed with POAG, PEXG, NTG, and pigment-dispersion glaucoma were eligible to participate in this study. Prior to this, the indication for trabeculectomy had to have been established using the EGS criteria [1]. Informed consent for the study was confirmed in writing by the patients.

In preparation for trabeculectomy, all preoperative IOP-lowering medication had been replaced by Dorzolamide 20 mg/mL + Timolol 5 mg/mL. preservative-free eye drops in the study eye for at least 28 days before the Triggerfish^®^ profile.

The best-corrected visual acuity (BCVA) had to be at least 20/200 or better in both eyes. Subjects presenting with any of the following criteria were not included in the trial:

- Subjects with contraindications for wearing contact lenses in the study eye.
- The diagnosis of secondary glaucoma in the study eye (according to EGS criteria).
- Previous intraocular surgery in the study eye ≤ three months ago.
- Previous refractive surgery in the study eye.
- Known sicca syndrome in the study eye.
- Known keratoconus in the study eye.
- Other abnormalities in the study eye that prevent safely wearing the contact lens sensor.
- A conjunctival or intraocular irritant condition in the study eye.
- Concurrent participation in another clinical trial.
- Previous IOP-lowering intervention in the study eye.
- Study participants performing shift work within the past three months.
- Transmeridian flight with a time difference ≥ six hours within the past three months.
- Study participants with a pacemaker.

**Figure 1:**
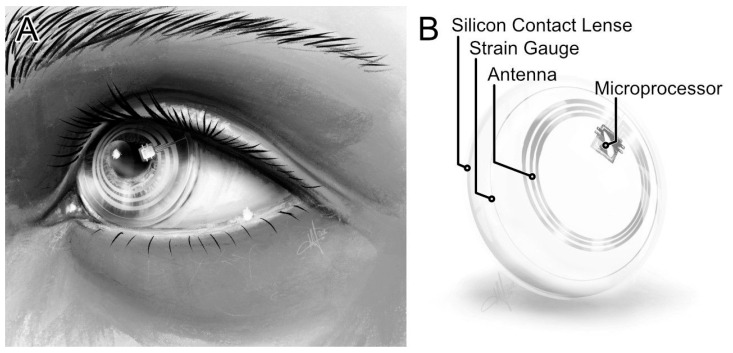
Contact lens sensor. (**A**) Placed CLS. (**B**) Components of the CLS.

The 24-h measurement before and after surgery was performed with the SENSIMED Triggerfish^®^ contact lens sensor (Figure 1).

The sensor consisted of a soft disposable contact lens with a diameter of 14.4 mm. The contact lens contained embedded strain gauges that registered ocular dimensional changes at the corneoscleral junction. These changes were mapped in the form of arbitrary units over the measurement period of 24 h. Several measurements were taken every five minutes for 30 s, from which the median was then calculated. This resulted in approximately 288 measurement points over 24 h. The measured data were transmitted via Bluetooth to a recorder, which the patient wore around the neck.

The primary endpoint of this study was the investigation of changes in amplitude, and the secondary endpoints were changes in the MESOR of modeled CLS data and the fluctuation in the raw CLS data. The CLS measurements were analyzed using a dual harmonic cosine function. The following formula could be used to estimate a fitted curve to analyze the 24-h CLS patterns (Equation (1)).

f(t) = M + A_1_∙cos(2π/τ t + ϕ_1_) + A_2_∙cos(2π/τ t + ϕ_2_) (1)

Equation (1): Dual harmonic cosine function. M = midline estimating statistic of rhythm (MESOR); A_1_ = first harmonic; A_2_ = second harmonic; ϕ_1_ = acrophase of first harmonic; ϕ_2_ = acrophase of second harmonic; τ = time period (24 h); t = time [hh:mm]

With the fitting function, the maximum, minimum, amplitude, and MESOR could also be calculated. These described the mean value of the rhythm-adjusted fitted curve. The maximum and minimum, as well as their time points, were calculated using the predict function of the software R [6]. The amplitude was calculated from half of the difference between the maximum and minimum. The circadian rhythm could thus be captured and evaluated. The variables of the fitting function were tested for significance for each data set. The raw data of the measured values were also used to calculate the diurnal fluctuations.

All parameters were compared before and after surgery. Besides changes in the MESOR and fluctuation in the raw CLS data, IOP, central corneal thickness (CCT), and safety and tolerability were evaluated as secondary endpoints. The best-corrected visual acuity (BCVA) and subjective refraction were assessed. The clinical condition of the study eye was evaluated before and after surgery with the modified ORA redness scale, and after surgery, the function of the filtering bleb was evaluated with the Würzburg Bleb Classification scale (WBCS) [7]. All patients underwent trabeculectomy according to the Mainz technique.

Statistics were generated and analysis of the data was performed using R statistical software (version R 4.3.1). Because of the size of the data set, descriptive statistics were generated first. If deemed appropriate, the Wilcoxon signed-rank test for dependent samples was applied.

## 3. Results

Nine patients, five with POAG, three with NTG, and one with PEXG, successfully completed this study. One participant had an incomplete data set after the second measurement, so this was not included in the analysis. Two other participants withdrew their consent because they were no longer interested, so nine study participants finished the study with complete data sets and were included in the evaluation. Three female and six male patients were studied, of whom three were between 40 and 50 years, five were between 60 and 70 years, and one participant was older than 70 years. The CLS wearing time was 23.9 ± 0.05 h for the measurement before surgery and 23.9 ± 0.03 h for the measurement after surgery, with at least 75 % of the measurement points recorded at both time points. Due to a retinal vein branch occlusion, one participant could be examined at the third visit only after 269 days of surgery. For the evaluation of this study, it seemed reasonable to include this study subject anyway despite that protocol deviation. The evaluation of the entire cohort showed a reduction in IOP (GAT) of 8.00 mmHg (−51 %) after surgery (*p* < 0.01) (Table 1).

**Figure 2:**
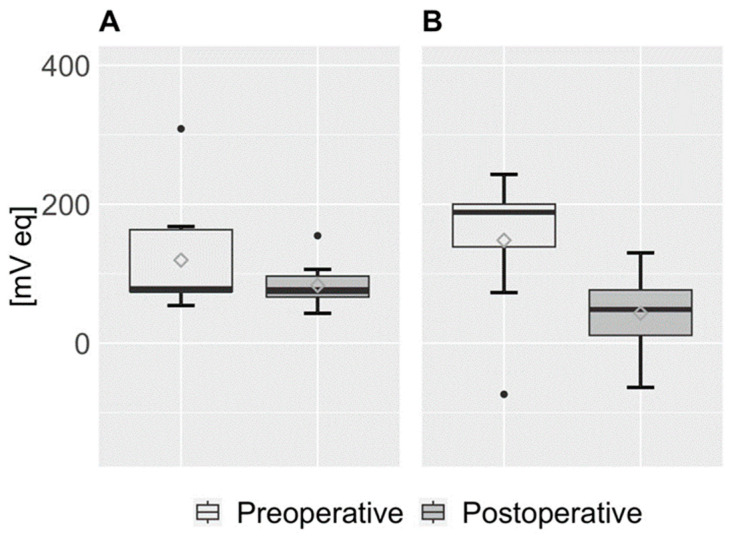
Boxplots of amplitude and MESOR. (**A**) Amplitude. (**B**) MESOR.

The statistics of the study population showed an amplitude of the CLS cosine analysis of 119.59 ± 82.40 mV eq with a median of 77.13 mV eq preoperatively and 83.34 ± 32.81 mV eq with a median of 75.66 postoperatively, which was not statistically significant (*p* > 0.05). The MESOR was 148.58 ± 96.37 mV eq preoperatively with a median of 188.42 mV eq and 43.06 ± 55.18 mV eq postoperatively with a median of 48.46 mV eq, which was statistically significant (*p* < 0.05) (Figure 2).

The distributions of the individual parameters of all study participants are shown pre- and postoperatively. The median is shown as a highlighted black line, and the mean is shown as a rhombus.

**Figure 3:**
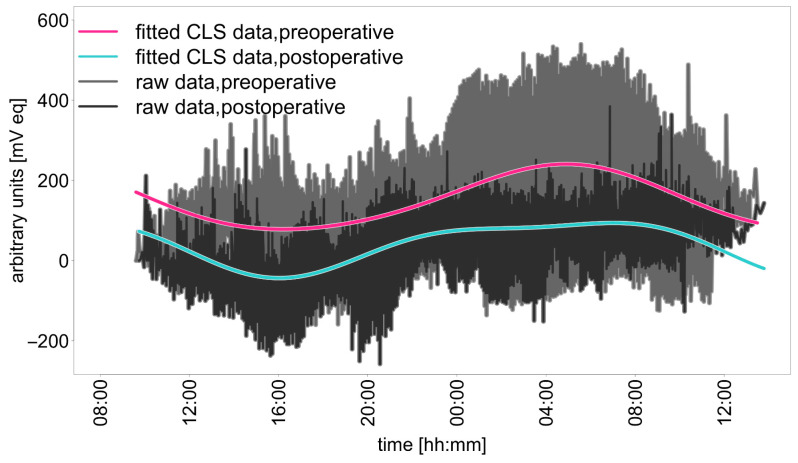
Raw data and curve fitted by Equation 1 for all study subjects before and after trabeculectomy.

We detected the acrophase, the nocturnal maximum value of the circadian rhythm, at night preoperatively in eight patients and postoperatively in seven patients. One participant in the study diagnosed with POAG had an acrophase at 3:51 a.m. before TE and at 2:09 a.m. after TE. Another POAG participant had an acrophase at 9:03 a.m., down from 4:11 a.m. before TE. In contrast, a participant with NTG had an acrophase at 9:32 a.m., which was later than the 10:15 p.m. acrophase observed prior to surgery. The remaining two individuals in this trial with NTG showed acrophases during nighttime hours, showing no significant changes before and after the surgical procedure. On average, we could observe the acrophase at 08:12 [hh:mm] before and at 08:13 [hh:mm] after surgery. In all patients, a periodicity in the compensation curve of 24 h could be observed, which corresponds to circadian rhythmicity. The graphical analysis also confirmed the above observation (Figure 2). The fitted CLS data of all patients show that not the amplitude but the MESOR statistically significantly decreased after surgery. In addition, it can be seen from the compensation curve that a flattened increase in the night phase occurred postoperatively until the maximum was reached (Figure 3).

The raw data of the contact lens sensor measurements were evaluated to assess the fluctuation during the day, night, and 24 h period pre- and postoperatively. A reduction in fluctuation of 66.34 mV eq during the day, 15.23 mV eq during the night, and 75.84 mV eq over the entire period was observed. None of these changes were statistically significant.

Preoperatively, the central corneal thickness was 517.89 ± 43.92 µm before the wearing period and 522.33 ± 49.98 µm after sensor removal. After surgery, the central corneal thickness was 512.44 ± 41.93 µm before wearing the sensor and 520.11 ± 46.38 µm after the removal of the sensor. There was no statistically significant change in the central corneal thickness before and after the period of wearing the CLS (Table 1).

Preoperatively, the best-corrected visual acuity differed before and after the wearing period. Preoperatively, a change in the mean logMAR visual acuity of 0.21 was observed. This change was statistically significant (*p* < 0.05). Postoperatively, no statistically significant change (*p* > 0.05) in logMAR was observed before and after the wearing of the sensor. Here, the difference in the mean values of the logarithmic visual acuity was 0.05.

The evaluation of subjective refraction revealed a change in the following parameters both preoperatively and postoperatively: sphere and cylinder. The change in these clinical parameters was not considered clinically relevant. Likewise, no statistical significance could be observed for the changes in the above-mentioned parameters (Table 1).

The redness of the eyelid changed only slightly pre- and postoperatively. Before the preoperative wearing period, one participant had no conjunctival erythema, seven showed mild conjunctival erythema, and one patient showed moderate conjunctival erythema. After the initial wearing period, conjunctival erythema was observed in all participants, with moderate conjunctival erythema being present most frequently (67%) (Table 2). Postoperatively, mild conjunctival erythema was observed in a lower proportion of participants (66%), with only one observation of one severe case of conjunctival erythema and two participants with none. After the postoperative wearing period, severe conjunctival erythema was observed in 77% of the participants and mild conjunctival edema in 66% most frequently. When corneal staining was assessed, mild staining was most frequently observed after the preoperative wearing period. Postoperatively, staining was observed in most participants before the wearing period (66%). After sensor removal, corneal staining was found in all study eyes, with six eyes with mild, two with moderate, and one eye with severe corneal staining. Intraocular inflammation was not observed in any participant at any time point.

A change in the frequency distribution was observed for all variables of the Wuerzburg Bleb Classification Scale compared to baseline. The most frequent findings were vascularization similar to the surrounding conjunctiva before the wearing period in 77%, no corkscrew vessels in 77%, microcysts lateral or medial to the filtering bleb in 44%, and no encapsulation in 88% of cases. After the wearing period, the proportion of study participants with the vascularization of the filtering bleb increased. The relative proportion of participants with vascularization similar to the surrounding conjunctiva was 55% and that with increased was 22%. After the removal of the sensor, corkscrew vessels were found in 33% of the filtering blebs. After the wearing period, microcysts could be found in all participants, with microcysts most frequently (55%) observed lateral or medial to the filtering bleb. After the wearing period, no encapsulation was found in 100% of the patients (Table 3).

Both before and after trabeculectomy, the CLS could be removed using suction or forceps. We could not detect any difference between the four study time points regarding the insertion or removal of the CLS. Most importantly, no study participant experienced hypotony or choroidal swelling after CLS removal after surgery. None of the study participants reported such severe impairment during the wearing period that they would have been forced to stop the measurement.

## 4. Discussion

The present study was designed to examine the change in CLS measurements before and after trabeculectomy in open-angle glaucoma patients. To the best of our knowledge, the feasibility of continuous measurements using the contact lens sensor after trabeculectomy has not been shown previously. This study also aimed to investigate the safety and feasibility of continuous monitoring using the CLS after trabeculectomy.

The initial plan was to examine 30 patients’ eyes. Unfortunately, due to the restrictions in force for a long time due to the coronavirus pandemic, this number could not be reached. Recruitment was stopped for all clinical trials for several months by the regulatory authorities in Germany.

In the present study, we found a statistically significant reduction of 105.08 mV eq in the MESOR in the measurements after trabeculectomy, compared to the preoperative measurements. The reduction in the amplitude was only descriptively lower (36.24 mV eq). A nonsignificant change in amplitude may indicate that diurnal fluctuations in CLS measurements persist even after trabeculectomy. This is in contrast to the reductions in diurnal fluctuations after pressure-lowering intervention described in the literature. To date, there is no known study that has carried out an examination using this contact lens sensor before and after trabeculectomy. It has been shown in several studies that a difference exists with respect to the measured values of the amplitude in healthy eyes versus those with glaucoma. Yong Woo Kim et al. showed that the amplitudes of the cosine-fit curve were significantly greater in NTG eyes than in healthy controls. [8]. In our study, only three participants with NTG were included. It might be possible that an isolated study of only NTG patients measured pre- and postoperatively with the contact lens sensor may present a similar result as in the study by Yong Wong. Pajic B et al. studied ten POAG patients before and after high-frequency deep sclerotomy (HFDS) with the contact lens sensor. A statistically significant decrease in the amplitude could not be shown here either [5]. In further studies, different types of glaucoma should be evaluated and analyzed in separate groups so that possible differences can be revealed.

The MESOR represents the mean value of a rhythm-adjusted fitted curve. One characteristic of the MESOR is that, unlike the mean, it better reflects the central tendency of a circadian rhythm [9]. It has already been shown in various studies that there is a certain relationship between conventional IOP measurements and the measurements using CLS [10,11,12]. A moderate correlation between IOP and CLS measurements in vivo has been demonstrated [12]. In addition, Leonardi et al. were able to demonstrate a linear positive correlation between an increase in IOP and CLS measurements using an experiment with enucleated pig eyes [13]. It could be assumed that the decrease in the MESOR represents an expression of the reduction in the averaged IOP over 24 h. A nonsignificant change in amplitude may indicate that diurnal changes in CLS measurements persist after trabeculectomy.

Based on this knowledge, further studies need to be performed to clearly demonstrate the relationship between IOP and CLS measurements.

The acrophase, the nocturnal maximum value of a circadian rhythm, was detected before surgery in eight participants between 21:00 p.m. and 08:00 a.m. Before surgery, only one study participant was observed to have an acrophase during the day. After surgery, two time points for the acrophase were observed during the day. The change in the average acrophase was not clinically relevant (08:12 [hh:mm] vs. 08:13 [hh:mm]). A previously listed study by Pajic et al. found different time points when examining ten POAG patients before and after HFDS using CLS. Here, the average acrophase time points were reduced from 03:24 a.m. preoperatively to 02:32 a.m. postoperatively [5]. Compared with our study, Pajic investigated a minimally invasive procedure and also a homogeneous study population. This could be a reason for the divergence in the acrophase.

Wasielica-Poslednik et al. observed a significant reduction in mean IOP values and IOP fluctuations in the day and at night at least 6 months after trabeculectomy [14]. Klink et al. observed a significant reduction in the maximum diurnal and nocturnal IOP after trabeculectomy [15]. In our study, the fluctuation descriptively decreased during the day by 66.34 mV eq, during the night by 15.23 mV eq, and over the entire period by 75.84 mV eq after trabeculectomy. In contrast to the findings of our study, a study by Tojo et al. observed a significant decrease in nocturnal fluctuation in CLS measurements in 24 patients suffering from PEXG after trabeculotomy but not in the daytime or with over a 24 h measurement [4]. This is particularly interesting since a study has shown that patients with PEX glaucoma have higher fluctuations than healthy individuals [16]. In addition, a study of PEX glaucoma patients before and after ab interno trabeculectomy (AIT) and AIT in combination with cataract surgery showed that the nocturnal fluctuation in the CLS profile was significantly reduced after both surgical procedures [17]. Due to the heterogeneous study population examined in our study and the small sample size, subgroup analysis was not performed and we only found descriptive but no significant differences, which may be due to the wide range of measurements.

We could not show a significant difference in the central corneal thickness (CCT) before and after the 24 h wearing periods. This is in contrast with a study by Tojo et al. with 22 subjects (11 healthy and 11 participants suffering from PEXG) that observed a significant increase in the CCT in the entire study population after wearing the contact lens sensor [16]. Hubanova et al. studied 20 healthy subjects using the contact lens sensor and looked at changes in the central corneal thickness. Here, the changes were determined overnight at two-hour intervals. It was shown that while wearing the sensor, the CCT increased by a maximum of 4% during the night. Hubanova also found that CCT almost reached its initial value after removal of the sensor [18].

Before surgery, a statistically significant decrease in the logarithmic visual acuity from 0.04 to 0.26 was found after the 24 h wearing period of the CLS. Postoperatively, however, no statistically significant difference was demonstrated before and after the 24 h wearing period. Smedt et al. also found a reduction in the BCVA after the wearing period in healthy subjects [19]. In their study, one explanation was that temporary corneal defects would be responsible for the deterioration in visual acuity. These results are comparable to our results of the preoperative use of the CLS, as an increase in corneal staining after the 24 h wearing period was observed. Nevertheless, despite a postoperative increase in corneal staining after the CLS wearing period, the change in visual acuity after the postoperative wearing period was without statistical significance. Furthermore, no statistically significant difference in refraction before and after the removal of the CLS could be observed.

After the pre- and postoperative wearing period, a change in the conjunctival erythema was observed most frequently. Even before the placement of the sensor, conjunctival erythema was observed pre- and postoperatively. After surgery, the erythema increased more than before surgery. Most frequently, moderate erythema was observed preoperatively and severe erythema was observed postoperatively. We also assessed corneal staining after both 24 h wearing periods. Corneal staining was found in all participants after the 24 h wearing periods pre- and postoperatively. Some authors have already investigated the clinical condition of the eye before and after wearing the CLS. In a study by Smedt et al., minor corneal staining was observed in nine study participants (90%) and generalized corneal staining in one participant (10%). Moreover, in the study, the fluorescein-positive impression of the limbal conjunctiva was demonstrated in 80% of the cases [17]. This finding could not be observed in our study. Mansouri et al. also found that 32 study participants (80%) experienced conjunctival hyperemia after the wearing period. This was mild in 30 participants and severe in 2 study participants. The majority of those affected showed hyperemia for only a short period of time. Persistent findings for three days were observed in only one patient [20].

Finally, the previous study results are only comparable with the preoperative wearing period. To date, no data are available for the use of the CLS after trabeculectomy. It can be assumed that besides the surgery itself, it is probable that the use of mitomycin C and 5-fluorouracil may also have an influence on the irritation state of the conjunctival epithelium. A limitation of our study is the lack of follow-up at defined time points after sensor removal. To better assess the course of the clinical condition of the eye after the wearing period, future work should include an extended follow-up period.

Filtering bleb was graded by the Wuerzburg Bleb Classification Scale according to Grehn [21]. Here, the four parameters of vascularization, corkscrew vessels, capsulation, and microcysts are examined. The first three parameters are considered negative, as they are associated with unfavorable filtering bleb development. However, the development or presence of microcysts is considered prognostically favorable [21]. In the present work, after the wearing period, we observed an increase in vascularization and corkscrew vessels in two participants, a decrease in capsulation in all participants, and an increase in microcysts in two participants.

It was already described by Caglar that absence of vascularization and corkscrew vessels, as well as present microcysts, are associated with good filtering bleb function [22]. A small increase in vascularization, a decrease in corkscrew vessels as well as encapsulation, and the increased presence of microcysts led to the assumption that a 24 h measurement after trabeculectomy using the CLS is possible and safe. A limitation of the present work is that the evolution of filtering bleb function in the longer term was not assessed. An interesting aspect would be whether a measurement with the contact lens sensor after trabeculectomy has an influence on long-term filtering bleb function.

A limitation of this study is the small number of study participants and the associated low statistical power. This could potentially influence the comparison of the amplitude more than the comparison of the MESOR. Larger study populations should be considered for investigating this issue.

Since sleeping position can have an influence on the course of the CLS measurement, information about this would be interesting. Likewise, information about the course of blood pressure during CLS measurement would be informative in the sense of long-term blood pressure measurement, as it has been shown that a drop in blood pressure at night is accompanied by an increase in CLS measurement values.

In addition to safety and tolerability, this study was the first to demonstrate the feasibility of a measurement using the contact lens sensor after trabeculectomy. Despite the fact that the sensor is more rigid than normal contact lenses, the sensor was successfully inserted and removed again in all cases. If it was not possible to remove the sensor using the suction cup provided, it was possible to remove the sensor in all cases with the aid of tweezers. To perform this, we carefully inserted the forceps between the edge of the sensor and the adjacent conjunctiva. This enabled us to neutralize the adhesive forces of the sensor and remove it.

## 5. Conclusions

A 24 h measurement using the CLS after trabeculectomy in open-angle glaucoma patients was feasible and safe. The evaluation of the data using a dual harmonic cosine function did not result in a statistically significant change in amplitude but did result in a statistically significant change in the MESOR after surgery. The non-significant change in amplitude and the visual inspection of the fitted curve for all study participants indicates that the modeled CLS data showed a comparable rhythm both before and after surgery (see Figure 3).

The statistically significant reduction in the MESOR with a non-significant change in amplitude after TE is a novel finding. It can be assumed that the significant reduction in the MESOR represents the reduction in the average IOP over 24 h. To date, no method of converting CLS data (mV eq) into IOP (mmHg) has been established. Given the statistical reduction in IOP and the MESOR after TE, further studies should investigate the relationship between IOP and CLS data. Developing a method to convert CLS data into IOP would provide a new approach to examining glaucoma patients and assessing the success of trabeculectomy. Additionally, in the future, CLS data may serve as a surrogate parameter for IOP.

Finally, we observed no clinically relevant effects on filtering bleb function and the clinical condition of the eye after the 24 h wearing period.

This study is the first to show that measurement with CLS after trabeculectomy is both safe and tolerable.

## Figures and Tables

**Table 1:** Changes in CCT, BCVA, refraction, IOP, CLS amplitude, and MESOR pre- and postoperatively (entire cohort).

	Preoperative			Postoperative		
	Before 24 h Wearing Period	After 24 h Wearing Period	*p*	Before 24 h Wearing Period	After 24 h Wearing Period	*p*
CCT (µm)	517.89 ± 43.92	522.33 ± 48.98	1	512.44 ± 41.93	520.11 ± 46.38	0.82
BCVA ^1^	0.04 ± 0.17	0.26 ± 0.25	0.01	0.16 ± 0.38	0.21 ± 0.19	0.40
Sphere	−0.86 ± 2.20	−1.31 ± 1.87	0.14	−0.72 ± 2.50	−0.25 ± 1.88	0.94
Cylinder	−0.64 ± 0.59	−0.72 ± 0.62	0.59	−0.75 ± 0.61	−1.03 ± 0.75	0.17
IOP (mmHg)	16.22 ± 4.94	16.44 ± 3.10	0.81	8.00 ± 2.60	8.26 ± 3.87	0.67
CLS ^2^ amplitude (mV eq)	-	119.59 ± 82.4		-	83.34 ± 32.81	0.3
CLS MESOR ^2^ (mV eq)	-	148.58 ± 96.37		-	43.06 ± 55.18	0.04
CLS fluctuation ^3^ (diurnal)	-	394.90 ± 110.98		-	328.56 ± 82.67	0.16
CLS fluctuation ^3^ (nocturnal)	-	268.39 ± 76.80		-	253.16 ± 40.98	0.82
CLS fluctuation ^3^ (24 h)	-	437.46 ± 135.38		-	361.62 ± 65.55	0.25

^1^ logMAR, ^2^ based on the modeled data using the dual harmonic cosine function, ^3^ raw CLS data.

**Table 2:** Changes in slit lamp examination before and after 24 h wearing period pre- and postoperatively.

Variable	Grade	Preoperative		Postoperative	
		Before 24 h Wearing Period	After 24 h Wearing Period	Before 24 h Wearing Period	After 24 h Wearing Period
Lid erythema, n (%)	0	6 (66)	6 (66)	7 (77)	6 (66)
	1	3 (33)	3 (33)	2 (22)	3 (33)
Lid edema, n (%)	0	8 (88)	9 (100)	7 (77)	8 (88)
	1	1 (11)	---	2 (22)	1 (11)
Conjunctival erythema, n (%)	0	1 (11)	---	2 (22)	---
	1	7 (77)	---	6 (66)	---
	2	1 (11)	6 (66)	---	1 (11)
	3	---	3 (33)	1 (11)	7 (77)
	4	---	---	---	1 (11)
Conjunctival edema, n (%)	0	9 (100)	4 (44)	7 (77)	1 (11)
	1	---	5 (55)	1 (11)	6 (66)
	2	---	---	1 (11)	2 (22)
Corneal staining, n (%)	0	3 (33)	---	3 (33)	---
	0.5	3 (33)	2 (22)	1 (11)	---
	1	2 (22)	6 (66)	3 (33)	6 (66)
	2	1 (11)	---	2 (22)	2 (22)
	3	---	1 (11)	---	1 (11)
Intraocular inflammation, n (%)	0	9 (100)	9 (100)	9 (100)	9 (100)

Lid erythema [0 = none (normal); 1 = mild (redness localized to a small region of the lid margin or skin]. Lid edema [0 = none (normal); 1 = mild (localized to a small region of the lid)]. Conjunctival erythema [0 = none (normal); 1 = mild (a flushed, reddish color predominantly confined to the palpebral or bulbar conjunctiva); 2 = moderate (a more prominent red color in the palpebral or bulbar conjunctiva); 3 = severe (definite redness in the palpebral or bulbar conjunctiva); 4 = very severe (severe redness in the palpebral and bulbar conjunctiva)]. Conjunctival edema [0 = none (normal); 1 = mild (slight localized swelling); 2 = moderate (moderate/medium localized swelling or mild diffuse swelling)]. Corneal staining [Grade 0 corresponds to no staining dots. Grade 0.5 was added compared to the normal Oxford scale. It corresponds to 1 to 3 staining dots. The other scores are the same as on the standard Oxford scale]. Intraocular inflammation [Slit beam = 0.3 mm wide; 1.0 mm long. 0 = none (no Tyndall effect)]. Conjunctival erythema was graded by the ORA Redness Scale (Grade 0–4) and corneal staining by the modified Oxford scale.

**Table 3:** Changes in the filtering bleb morphology graded by the WBCS according to Grehn [7].

Variable	Grade	Before 24 h Wearing Period	After 24 h Wearing Period
Vascularization, n (%)	3	2 (22)	2 (22)
	2	7 (77)	5 (55)
	1	---	2 (22)
Corkscrew vessels, n (%)	3	7 (77)	6 (66)
	2	2 (22)	1 (11)
	1	---	2 (22)
Microcysts, n (%)	3	3 (33)	3 (33)
	2	4 (44)	5 (55)
	1	1 (11)	1 (11)
	0	1 (11)	---
Encapsulation, n (%)	3	8 (88)	9 (100)
	2	1 (11)	---

Vascularity [3 = avascular; 2 = similar to adjacent conjunctiva; 1 = increased; 0 = massive]. Corkscrew vessels [3 = none; 2 = in one third; 1 = in two thirds; 0 = entire bleb]. Microcysts [3 = entire bleb; 2 = lateral or medial of flap; 1 = over scleral flap; 0 = none]. Encapsulation [3 = none; 2 = in one third; 1 = in two thirds; 0 = entire bleb]. Changes in the filtering bleb can be assessed according to the variables described by Grehn without permission.

## Data Availability

The raw data supporting the conclusions of this article will be made available by the authors on request.
